# A low aromatic amino-acid diet improves renal function and prevent kidney fibrosis in mice with chronic kidney disease

**DOI:** 10.1038/s41598-021-98718-x

**Published:** 2021-09-28

**Authors:** Christophe Barba, Bérengère Benoit, Emilie Bres, Stéphanie Chanon, Aurélie Vieille-Marchiset, Claudie Pinteur, Sandra Pesenti, Griet Glorieux, Cécile Picard, Denis Fouque, Christophe O. Soulage, Laetitia Koppe

**Affiliations:** 1 CarMeN Laboratory, INSA-Lyon, INSERM U1060, INRA, Univ. Lyon, Université Claude Bernard Lyon 1, Rhône, Oullins France; 2 Department of Nephrology and Nutrition, Centre Hospitalier Lyon SUD, Hospices Civils de Lyon, Chemin du Grand Revoyet, 69495 Pierre Bénite, France; 3 Nephrology Section, Department of Internal Medicine and Pediatircs, Ghent University Hospital, Ghent, Belgium; 4 Centre de Biologie et Pathologie Est, Hospices Civils de Lyon, Bron, France

**Keywords:** Nephrology, Kidney diseases

## Abstract

Despite decades of use of low protein diets (LPD) in the management of chronic kidney disease (CKD), their mechanisms of action are unclear. A reduced production of uremic toxins could contribute to the benefits of LPDs. Aromatic amino-acids (AA) are precursors of major uremic toxins such as p-cresyl sulfate (PCS) and indoxyl sulfate (IS). We hypothesize that a low aromatic amino acid diet (LA-AAD, namely a low intake of tyrosine, tryptophan and phenylalanine) while being normoproteic, could be as effective as a LPD, through the decreased production of uremic toxins. Kidney failure was chemically induced in mice with a diet containing 0.25% (w/w) of adenine. Mice received three different diets for six weeks: normoproteic diet (NPD: 14.7% proteins, aromatic AAs 0.019%), LPD (5% proteins, aromatic AAs 0.007%) and LA-AAD (14% proteins, aromatic AAs 0.007%). Both LPD and LA-AAD significantly reduced proteinuria, kidney fibrosis and inflammation. While LPD only slightly decreased plasma free PCS and free IS compared to NPD; free fractions of both compounds were significantly decreased by LA-AAD. These results suggest that a LA-AAD confers similar benefits of a LPD in delaying the progression of CKD through a reduction in some key uremic toxins production (such as PCS and IS), with a lower risk of malnutrition.

## Introduction

Because nutritional status often becomes unbalanced during the course of chronic kidney disease (CKD), nutritional intervention is a mandatory component of the management of patients with CKD^1^. It has long been recognized that high protein intake has a deleterious impact on renal function in CKD patients, by increasing glomerular filtration rate (GFR), leading to progressive glomerular sclerosis^2,3^. Hence, a low protein diet (LPD) (i.e. 0.6 to 0.8 g/kg/day) is now commonly recommended for non-hemodialysis CKD patients, decreasing kidney workload and slowing renal function decline^4^. For decades, various health benefits have been attributed to protein restriction in patients with CKD, such as favorable metabolic effects, reduction of proteinuria and uremic symptoms and improvement in insulin-sensitivity^5^. One of the hypotheses put forward to explain these effects is a potential decrease in protein-derived uremic toxin concentrations, improving renal and metabolic parameters and leading to better uremia control^6^. However, if LPDs were shown to efficiently delay CKD progression, the underlying mechanisms remain poorly understood. Besides, this nutritional strategy has several drawbacks, such as a poor compliance and a potential risk of energy wasting^7^.

p-Cresyl sulfate (PCS) and indoxyl sulfate (IS) are protein-bound uremic toxins produced by the gut microbiota^8^. Previous studies showed that CKD patients microbiota exhibited a loss of biodiversity and a decrease in the number of bacterial operational taxonomic units^9^. Indeed, dysregulation of intestinal microbiota, i.e. dysbiosis, could contribute to the generation of uremic toxins^10^. Indole and p-cresol the precursors of IS and PCS are produced by intestinal bacteria from the fermentation of aromatic amino acids, tryptophan and tyrosine (and also to a limited extent phenylalanine), respectively^11^. A strong association has been demonstrated between accumulation of PCS and cardiovascular damages. PCS concentrations are associated with the rise of inflammatory markers (e.g.: IL-6) and the increase in oxidative stress, through the production of reactive oxygen species^12,13^. It has also been shown that IS levels are negatively correlated with the GFR and contribute to the progression of CKD^12^. Consequently, lowering uremic toxin levels in CKD patients appears to be a relevant strategy to prevent cardiovascular events and deterioration of kidney function.

Several studies clearly suggest that variations in protein quantity and/or quality induce marked changes in metabolism parameters, and substantial progress has been made in identifying potential mechanisms underlying these effects^14^. In particular, evidence suggests that enriching a diet with plant-based proteins (vs animal proteins) could reduce the risk of developing type 2 diabetes mellitus and metabolic diseases^15^. In the CKD area, recent publications suggest that plant based diet improve renal outcomes^16,17^.

The aim of the present study was to determine whether a normoproteic diet (NPD) selectively deprived in aromatic amino acids (i.e. tyrosine, tryptophan and phenylalanine), could be as beneficial as LPD in an experimental model of CKD. To test our hypothesis, we addressed two specific aims: i) To decipher whether a low aromatic amino acid diet (LA-AAD) could efficiently reduce renal fibrosis and inflammation in CKD mice ii) To assess if a LA-AAD is efficient to reduce IS and PCS production in CKD mice.

## Results

### Low aromatic amino acid diet does not alter food intake and body composition in CKD mice

In order to study in CKD mice, the specific effects of reducing dietary intake of three aromatic amino acids on uremic toxins production and renal function, we designed and constructed an amino acid-defined NPD modeled on a 14% protein diet used as standard (AO4 diet). We also investigated two additional diets: a LPD with a global decrease in amino acids content, in which 5% of calories were derived from amino acids through a uniform reduction of every amino acid in the NPD diet; and a LA-AAD in which all aromatic amino acids were reduced by two-thirds to match the levels of the LPD, while all other amino acids were kept at the level of the NPD. All three diets were isocaloric with identical levels of dietary fat; the exact formulations of these 3 experimental diets are provided in Table 1.

**Table 1:** Food composition and energy value of the different diets.

	NPD	LPD	LA-AAD
Starch (g/kg)	394.4	520.5	407.8
Maltodextrin (g/kg)	142.0	142.0	142.0
Sucrose (g/kg)	110.0	110.0	110.0
Soya oil (g/kg)	70.0	70.0	70.0
Cellulose (g/kg)	50.0	50.0	50.0
Choline bitartrate (g/kg)	2.5	2.5	2.5
**Aminoacid composition g/kg**			
Alanine (Ala)	4.93	1.62	4.93
Arginine (Arg)	6.08	1.98	6.08
Aspartic Acid (Asp)	11.56	3.78	11.56
Cysteine (Cys)	3.69	1.23	3.69
Glutamic Acid (Glu)	36.63	12.09	36.63
Glycine (Gly)	3.05	1.00	3.05
Histidine (His)	5.00	1.63	5.00
Isoleucine (Ile)	9.02	2.95	9.02
Leucine (Leu)	15.29	4.99	15.29
Lysine (Lys)	13.41	3.68	13.41
Methionine (Met)	4.98	1.65	4.98
Phenylalanine (Phe)*	**8.43**	**2.75**	**2.74**
Proline (Pro)	18.02	5.95	18.02
Serine (Ser)	10.15	3.33	10.15
Threonine (Thr)	7.09	2.33	7.09
Tryptophan (Trp)*	1.88	0.62	0.59
Tyrosine (Tyr)*	9.31	3.07	3.07
Valine (Val)	11.56	3.78	11.56
**Protein (%)**	**14.7**	**5.0**	**14.0**
Carbohydrates (%)	13.8	13.8	13.8
Fat (%)	7.1	7.2	7.1
Fibers (%)	3.6	3.6	3.6
Starch (%)	47.6	59.0	48.8
Minerals (%)	2.3	2.3	2.3
Energy density (kcal/g)	3.89	3.84	3.89
**Aromatic amino acids (mg/g)**	**19.62**	**6.44**	**6.40**

Data are expressed as percentage for food composition and kcal/g for energy value.

*NPD* Normoproteic diet, *LPD* Low protein diet, *LA-AAD* Low aromatic amino-acid diet.

*Aromatic amino acids including tyrosine, tryptophan and phenylalanine are displayed in bold. Note that the low aromatic AA diet concentration of aromatic amino acids was similar to that low protein diet.

**Figure 1:**
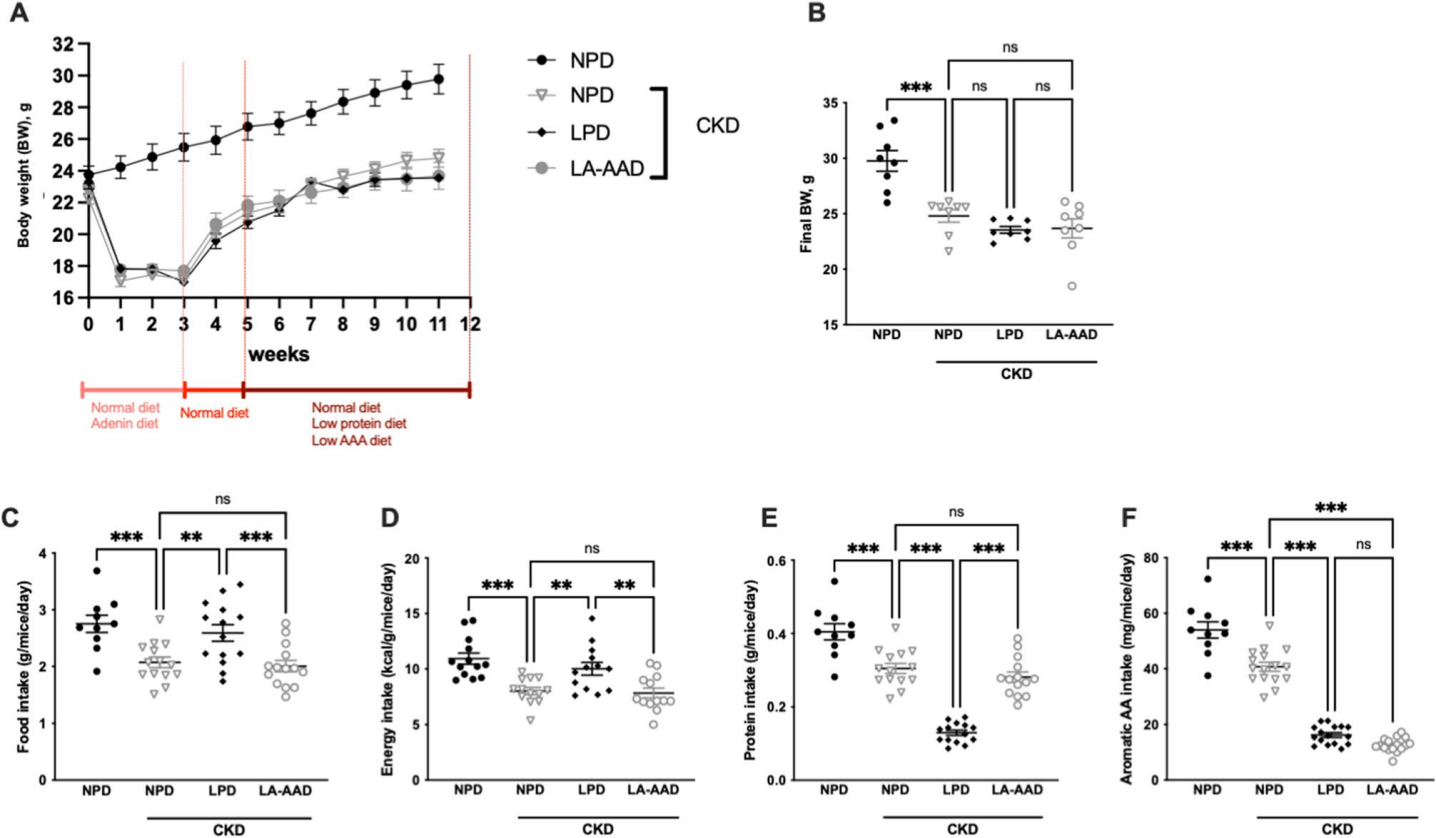
Food, energy, protein and aromatic amino acids intakes according to each specific diet and renal condition. Body weight evolution (**A**), final body weight (**B**), food intake (**C**), energy intake (**D**), protein intake (**E**) and aromatic amino-acid intake (**F**) in control mice fed with normoproteic diet (NPD) and chronic kidney disease (CKD) mice fed with NPD, low protein diet (LPD) or low aromatic amino-acid diet (LA-AAD). Data are presented as mean ± SEM for n = 10–13 animals in each group. *p < 0.05, **p < 0.01, ***p < 0.001 versus CKD-NPD; (ANOVA and Dunnett post hoc test).

We first ensured that LA-AAD diet had no significant impact on food intake and body composition in the control group. Energy intake was similar in all diets. Control LPD mice gained less weight than mice on NPD or LA-AAD over the course of six weeks as previously described^18^ (*data not shown*). Control mice fed with NPD exhibited a higher body weight compared to CKD mice. However, no significant difference in body weight was noticed between CKD mice fed with NPD, LPD or LA-AA diet (Fig. 1A,B). We therefore proceeded to use control mice with NPD as baseline for investigation of the specific contribution of reduced dietary aromatic acids in CKD mice.

Dietary intakes are summarized in Fig. 1. Food intake (Fig. 1C) and energy intake (Fig. 1D) were lower in CKD-NPD mice, compared to the control group. CKD mice fed with LPD had a higher food and energy intake compared to CKD-NPD. However, there was no difference on food intake between CKD-NPD and CKD-LA-AAD. The average protein intake was 57% (*p* < 0.001) and 54% (*p* < 0.001) lower in CKD-LPD group, compared to CKD-NPD and CKD-LA-AAD groups, respectively (Fig. 1E). Aromatic amino-acid intakes were significantly lower in both LPD (*p* < 0.0001) and LA-AAD (*p* < 0.0001) groups, compared to mice fed with NPD (Fig. 1F). There was no statistical difference in aromatic amino-acid intake between CKD-LPD and CKD-LA-AAD groups (Fig. 1D).

Biometric data and organ weights are presented in Table 2. At the end of the experimental study, the body weights of all CKD mice were lower than those of control mice. However, among CKD mice, no statistical difference in body weight was observed between the 3 specific diets. CKD-LPD had a slightly higher fat deposition that can be explained by the higher food intake but CKD-NDP and CKD-LA-AAD exhibited similar lean and fat mass. LPD and LA-AAD did not affect the nutritional status and surprisingly, CKD mice treated with LA-AAD exhibited increase albuminemia. (Table 3) Kidney weights were lower in CKD compared to control groups. The kidney weight of CKD-LPD mice was significantly lower than in CKD-NPD. There was no difference of kidney weight between and CKD-LA-AAD groups and CKD-NPD.

**Table 2:** Biometric data.

Variable	Control mice	CKD mice		
Diet	NPD	NPD	LPD	LA-AAD
N	9	11	8	13
**Biometric data**				
BW (g)	29 ± 1***	22 ± 0.1	20 ± 1	21 ± 0.1
BL (cm)	9.5 ± 0.1***	8.7 ± 0.1	8.6 ± 0.1	8.6 ± 0.1
**White adipose tissue weights, mg/10 g BW**				
Total WAT	297 ± 24***	208 ± 14	333 ± 14*	228 ± 9
rWAT	55 ± 6***	33 ± 2	67 ± 3*	35 ± 2
eWAT	242 ± 18***	175 ± 12	266 ± 12*	194 ± 7
**Organ weights, mg/10 g BW**				
Heart	45 ± 1***	55 ± 3	50 ± 1	51 ± 1
Gastrocnemius	48 ± 2*	57 ± 2	50 ± 2	54 ± 1
Liver	413 ± 8	395 ± 14	347 ± 8	374 ± 11
Kidneys	119 ± 2***	86 ± 3	54 ± 2*	76 ± 1

Data are expressed as mean ± SEM.

*BL* Body length, *BW* Body weight, *NPD* Normoproteic diet, *LPD* Low protein diet, *LA-AAD* Low aromatic amino-acids diet, *WAT* White adipose tissue, *eWAT* Epidydimal WAT, *rWAT* Retroperitoneal WAT.

*p < 0.05; ***p < 0.001 versus CKD-NPD (ANOVA and Dunnett post hoc test).

**Table 3:** Biochemical data.

Variable	Control mice	CKD mice		
	NPD	NPD	LPD	LA-AAD
Fasting glucose (mmol/L)	4.1 ± 0.2	4.3 ± 0.2	3.2 ± 0.1*	4.5 ± 0.1
Fed glucose (mmol/L)	8.2 ± 0.4	8.8 ± 0.5	6.8 ± 0.5	8.1 ± 0.5
Total cholesterol (mmol/L)	4.0 ± 0.3	3.3 ± 0.4	3.6 ± 0.1	3.9 ± 0.2
Triglycerides (mmol/L)	2.3 ± 0.2	2.7 ± 0.2	1.5 ± 0.2*	3.0 ± 0.2
Plasma proteins (g/L)	46.7 ± 1.9	45.7 ± 1.5	47.8 ± 4.4	46.9 ± 1.9
Serum albumin (g/L)	15.8 ± 0.8	18.9 ± 0.5	18.1 ± 0.5	21.1 ± 0.8
Diuresis (mL/24 h)	1.2 ± 1.0***	3.2 ± 1.6	2.0 ± 0.9*	1.9 ± 0.9*

Data are expressed as mean ± SEM for n = 5–12 animals in each group.

*NPD* Normoproteic diet, *LPD* Low protein diet, *LA-AAD* Low aromatic amino-acids diet, *CKD* Chronic kidney disease.

*p < 0.05; **p < 0.01; ***p < 0.001 versus CKD-NPD (ANOVA and Dunnett post hoc test).

### Specific reduction of dietary aromatic amino acids attenuates kidney injury in CKD mice

**Figure 2:**
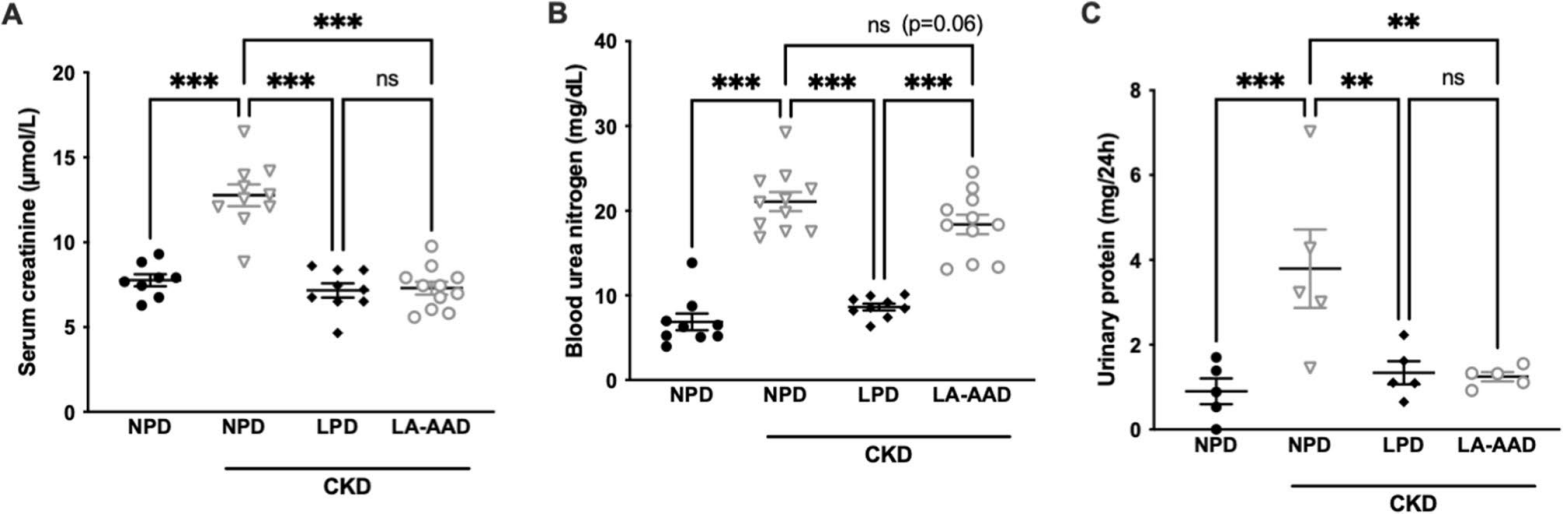
Kidney function and urinary protein excretion in control and CKD mice. Serum creatinine (**A**), blood urea nitrogen (**B**) and urinary proteins (**C**) in control and CKD mice fed with normoproteic diet (NPD), low protein diet (LPD) or low aromatic amino-acid diet (LA-AAD). Data are expressed as mean ± SEM for n = 9–11 animals in each group. Proteinuria was only measured on 5 animals per groups. *p < 0.05, **p < 0.01, ***p < 0.001 versus CKD-NPD; (ANOVA and Dunnett post hoc test).

**Figure 3:**
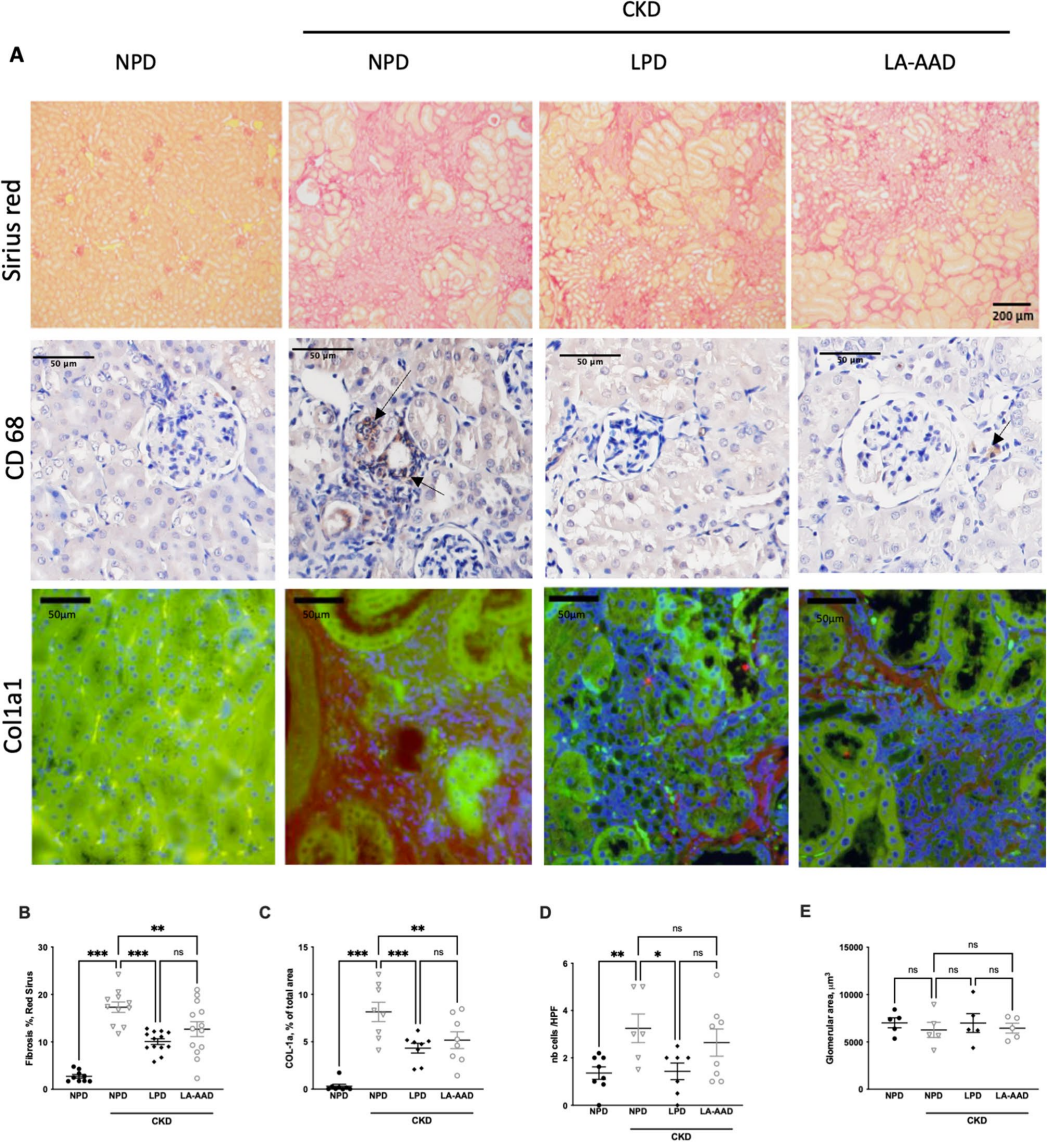
Evaluation of kidney fibrosis, glomerulosclerosis and kidney inflammation in control and CKD mice. (**A**) Representative images of kidney sections stained with Sirius Red viewed under bright field × 10. Scale bar represents 200 µm. Immunohistochemistry staining for CD68 and immunofluorescence staining for Col1a1. Scale bar represents 50 µm. (**B**) Sirius red morphometric and (**C**) Col1a1 evaluation in control and CKD mice treated with normoproteic diet (NPD), low protein diet (LPD) and low aromatic amino-acid diet (LA-AAD). (**D**) Tubular cell damage scores obtained by CD68 quantification (E) Glomerular area was quantified using ImageJ software and expressed as glomerular volume. CKD: chronic kidney disease. Data are expressed as mean ± SEM for n = 9–13 animals in each group. **p < 0.01, ***p < 0.001 versus CKD-NPD; (ANOVA and Dunnett post hoc test). *nb* Numbers.

Eight weeks after the end of the adenine diet, circulating creatinine and urea levels in CKD-NPD were in the same range as those observed in moderate uremic patients (Fig. 2A,B). Serum creatinine levels were significantly decreased in CKD-LPD and CKD-LA-AAD compared to CKD-NPD (*p* < 0.0001), (Fig. 2A). Blood urea nitrogen levels (Fig. 2B) were lower in CKD-LPD as compared with that with NPD, but no significant difference was observed between CKD-NPD and CKD-LA-AAD. CKD-LA-AAD and CKD-LPD exhibited a similar reduction of urinary protein excretion reaching levels comparable to the NPD group (*p* < 0.05) (Fig. 2C). Fibrosis as measured with sirius-red positive areas or through collagen 1 (COL-1) immunofluorescence staining, were significantly lower in CKD-LPD and CKD-LA-AAD groups compared to CKD-NPD group (10 ± 1% and 13 ± 2% vs 17 ± 1%, *p* < 0.0001 and *p* = 0.01 for Sirius-red and 4.3 ± 1.4% and 5.2 ± 2.5% vs 8.1 ± 2.9%, *p* < 0.001 and *p* = 0.001 for COL-1) (Fig. 3A–C). There was no statistical difference in terms of percentage of fibrosis area between LPD and LA-AAD fed mice (Fig. 3B,C). Renal inflammation as evidenced by CD68 immunohistochemical staining is increased in CKD mice. LPD and LA-AAD almost completely abrogated the renal inflammation in CKD mice (Fig. 3D). Because adenine model mainly induces tubulointerstitial damages^19^, we failed to report any difference in glomerular volume (Fig. 3E) and no globally sclerotic glomeruli could be observed (data not shown).

**Figure 4:**
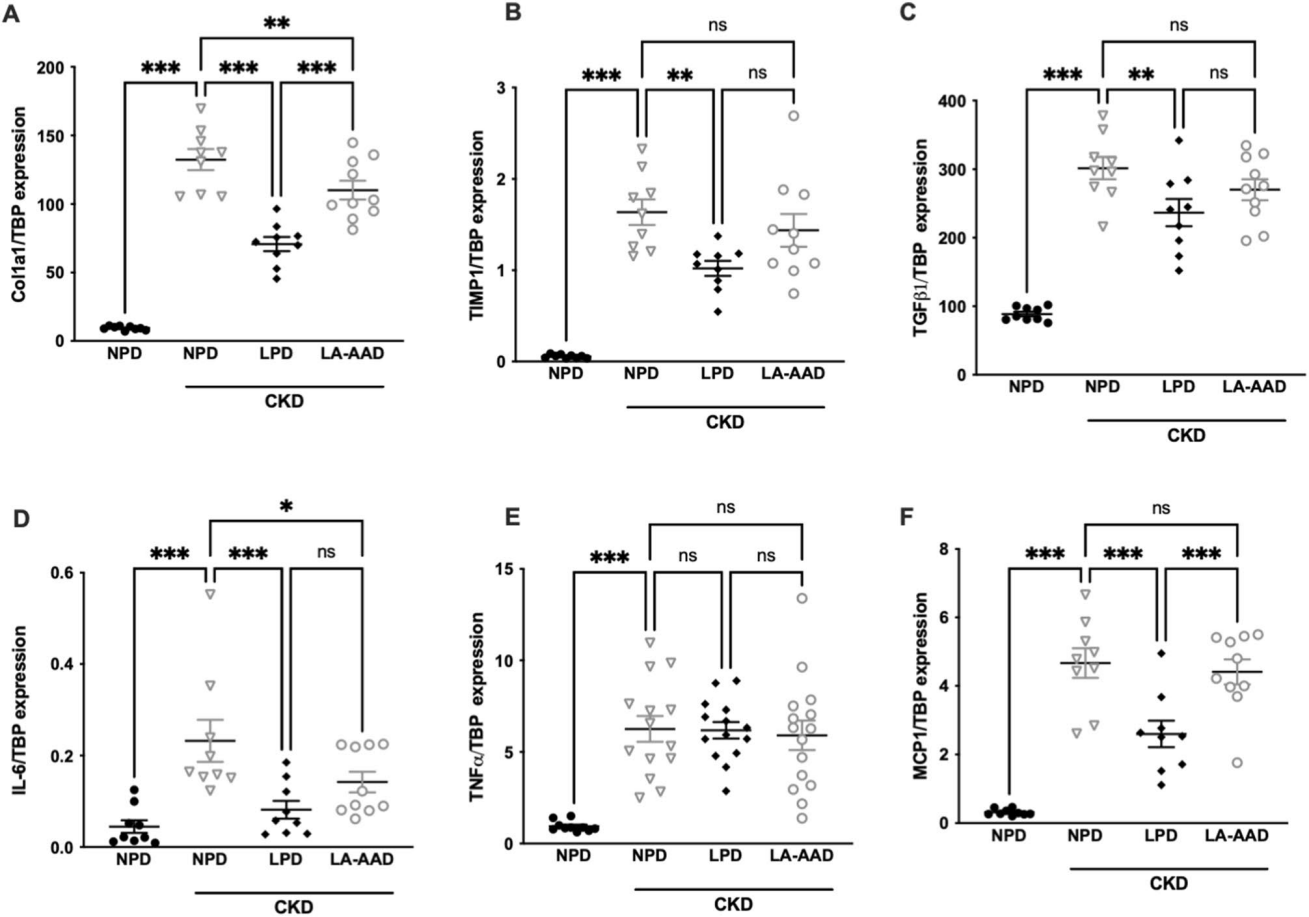
Gene expression of fibrosis and pro-inflammatory markers in kidney. Effects of normoproteic diet (NPD), low protein diet (LPD) and low aromatic amino-acid diet (LA-AAD) on relative mRNA expression of (**A**) Col1a1 (collagen alpha-1 type 1), (**B**) TIMP1 (Tissue Inhibitor of Metalloproteinase 1), (**C**) TGFβ1 (Transforming Growth Factor beta 1), (**D**) IL-6 (Interleukin 6), (**E**) TNFα (Tumor Necrosis Factor alpha) and (**F**) MCP-1 (Monocyte Chemoattractant protein 1) in control and CKD mice . TBP (TATA-Box Binding Protein) was used as reference gene to normalize the results. For gene expression, the relative fold gene expression level for each sample was calculated using the 2^−∆∆Ct^ method. Results are expressed as the ratio of target mRNA to TBP mRNA. Data are expressed as mean ± SEM for n = 9–10 animals in each group. *p < 0.05, **p < 0.01, ***p < 0.001 versus CKD-NPD; (ANOVA and Dunnett post hoc test).

We further conducted an analysis of gene expression of kidney fibrosis markers and pro-inflammatory cytokines, using real time PCR (Fig. 4). Quantitative PCR indicated a significant reduction in the expression of renal fibrosis-related genes (*Col1a1* (collagen alpha-1 type 1), *TIMP1 (*Tissue Inhibitor of Metalloproteinase 1), *TGFβ1 *(Transforming Growth Factor beta 1)) and inflammatory cytokines (*IL-6 (Interleukin 6)*, *TNFα (Tumor Necrosis Factor alpha),* and *MCP-1* (Monocyte Chemoattractant protein 1)) in the kidneys of the CKD-LPD group compared with the CKD-NPD group. The expression of *Smad3* (Small Mothers Against Decapentaplegic 3) was not significantly different between groups (*data not shown*). LA-AAD also improved the progression of renal fibrosis and local inflammation. In comparison with the CKD-NPD, *Col1A1* and *IL-6* expression was reduced with LA-AAD. Expressions of *TIMP1, TGFβ1*, *TNFα* and *MCP-1* mRNA were not significantly different between CKD-NPD and CKD-LA-AAD. The tubular injury was evaluated by *Kim 1*- mRNA (kidney injury molecule-1) expression (a common marker of tubular injury), with LA-AAD improved *Kim-1* expression compared to CKD mice fed with NPD or LPD (Supplementary Fig. 1).

### Low aromatic amino-acid diet has no impact on metabolic parameters

Fasting glycaemia was lower in CKD mice fed with the LPD and no significant difference were observed between CKD-mice fed with the NPD and LA-AAD. (Table 3). In good agreement, CKD mice fed the LPD diets for six weeks again showed improved glucose tolerance, but mice fed the LA-AAD diet showed no improvement in glucose tolerance (Supplementary Fig. 2).

### Restriction in dietary aromatic amino acids decreases free uremic toxins concentration

**Figure 5:**
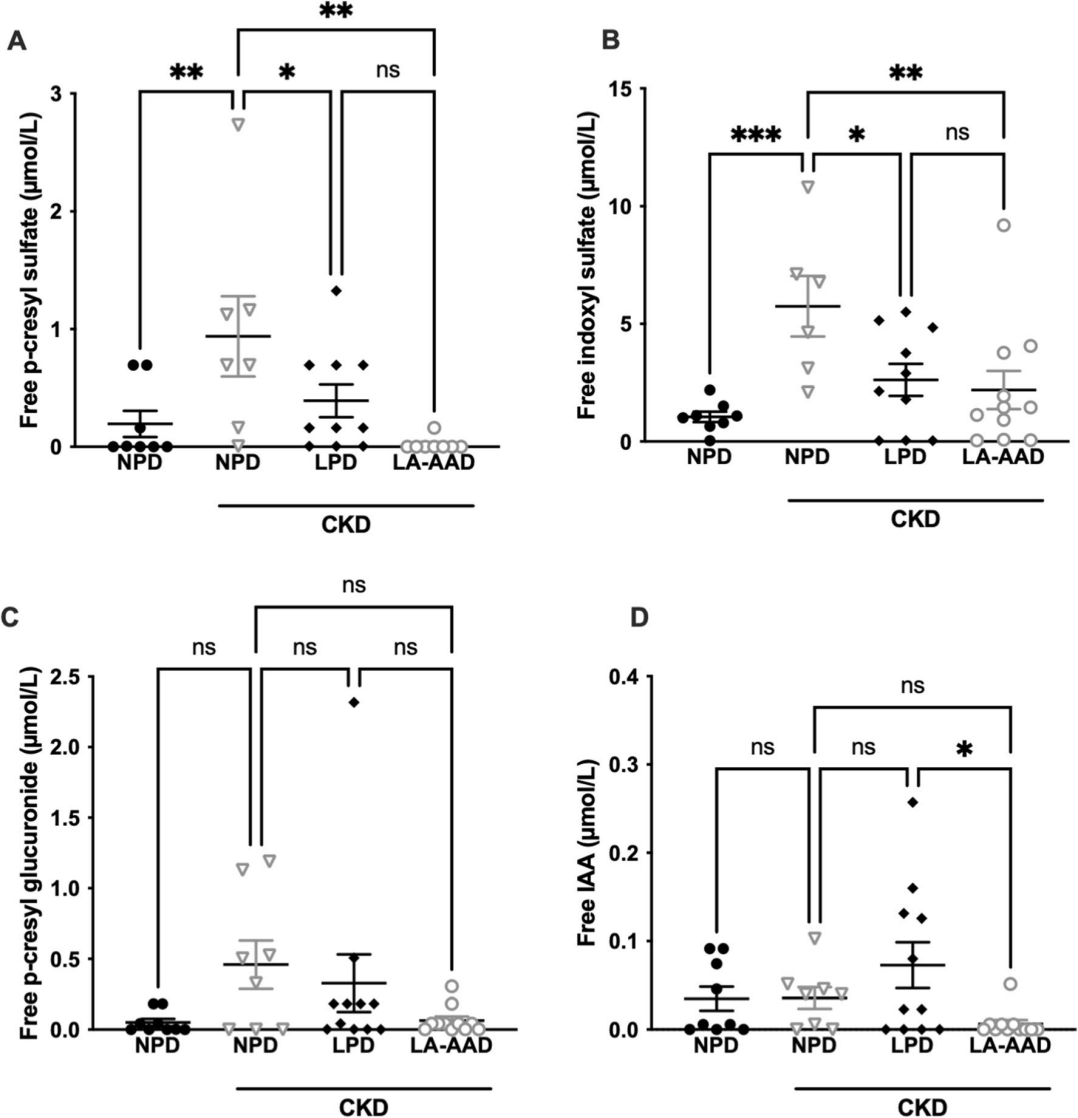
Free uremic toxin concentrations in control and CKD mice. Effects of normoproteic diet (NPD), low protein diet (LPD) and low aromatic amino-acid diet (LA-AAD) on free (**A**) p-cresyl sulfate, (**B**) indoxyl sulfate, (**C**) p-cresyl glucuronide and (**D**) indole 3-acetic-acid (IAA) concentrations in control and CKD mice. Data are expressed as mean ± SEM for N = 8–11 animals in each group. *p < 0.05, **p < 0.01, ***p < 0.001 versus CKD-NPD; (ANOVA and Dunnett post hoc test). *CKD* Chronic kidney disease.

To elucidate the mechanism of the renoprotective effect induced by LA-AAD and LPD, we investigated the effect of these diets on uremic toxins production. Total uremic toxin levels are presented in Table 4 and free uremic toxin concentrations in Fig. 5. 3Total PCS, total p-cresyl glucuronide (PCG), and total IS were increased in CKD mice compare to control mice but we did not observe any difference between CKD mice treated with different diets. We found no statistical differences for total indole-3-acetic-acid (IAA) concentrations between groups**.** CKD mice fed with LA-AAD diet exhibited significantly lower free PCS (*p* < 0.01) and free IS (*p* < 0.05) levels while LPD only modestly but significantly decreased the concentration of these uremic toxins in comparison to NPD. Only CKD mice fed with LA-AAD had a higher percentage of protein binding (%PB) of IS and PCS (Supplementary Fig. 3A,B). We failed to find any significant difference between groups for free PCG and free IAA.

**Table 4:** Serum concentration of total uremic toxins (µmol/L).

Variable	Control mice	CKD mice		
	NPD	NPD	LPD	LA-AAD
PCS	0.6 ± 0.2**	4.5 ± 0.9	3.6 ± 1.1	2.5 ± 0.7
PCG	0.1 ± 0.1*	0.6 ± 0.3	0.6 ± 0.3	0.2 ± 0.1
IS	8.2 ± 1.2***	37.5 ± 3.1	28.6 ± 2.3	42.6 ± 4.7
IAA	0.2 ± 0.1	0.2 ± 0.1	0.3 ± 0.1	0.3 ± 0.1
HA	290 ± 52	284 ± 48	298 ± 38	276 ± 14
Uric acid	64.5 ± 9.8	87 ± 19	78 ± 25	64 ± 13

Data are expressed as mean ± SEM.

*CKD* Chronic kidney disease, *PCS* P-cresyl sulfate, *PCG* p-cresyl glucuronide, *IS* Indoxyl sulfate, *IAA* Indole-3-acetic-acid, *HA* Hippuric acid, *NPD* Normoproteic diet, *LPD* Low protein diet, *LA-AAD* Low aromatic amino-acids diet.

*p < 0.05; **p < 0.01; ***p < 0.001 versus CKD-NPD (ANOVA and Dunnett post hoc test).

## Discussion

In this study, we demonstrated first that a LA-AAD exhibited a similar reno-protective effect as a LPD without the need of reducing total protein content. Indeed, the concomitant observation of kidney fibrosis, serum creatinine and urinary protein improvement provides support that reducing aromatic amino acids is efficient to metabolically mimic the action of a LPD. It is recognized from previous studies^7^ that patients under a LPD are associated with both protein and energy intake declines and there were small but significant declines in various indices of nutritional status at the beginning of the diet introduction^4,20^. This experiment was not designed to explore the nutritional status in the different groups. Nevertheless, we failed to find any difference in weight in CKD groups, neither for the LPD group nor for the LA-AAD group.

In this study, we showed secondly that a specific restriction in the intake of aromatic amino acids lowered some key uremic toxin (such as PCS and IS) concentrations and mitigated inflammation that play major roles in the progression of renal damage. Surprisingly, in our CKD experimental model, LPD was able to decrease only to a limited extent the free uremic toxin concentrations, while total uremic toxin concentrations were not significantly different between each CKD diet groups. Black & al reported in a longitudinal study with 30 non-dialysis CKD patients (stage 3–4) a favorable effect of LPD to improve renal function and decrease uremic toxins concentrations after six months of nutritional intervention^6^. However, they only found a significant effect on total PCS values, but neither on IS nor on IAA serum concentration. It is very likely that lower uremic toxin concentrations could have been reached using either very low protein diet (VLPD), or aromatic amino acid corresponding concentrations. Indeed, a recent study showed that VLPD was effective to beneficially modulate gut microbiota, improving intestinal permeability and reducing serum levels of total and free IS and PCS in CKD patients^21^. We cannot exclude that in the present study, the composition of diet by itself could have altered intestinal microbiota and uremic toxins production. For example, the total amount of starch is higher in LDP and LA-AAD than in NPD. Starch has recently been described as a prebiotic that promotes proliferation of some gut bacteria such as *Bifidobacteria* and *Lactobacilli,* increases the production of metabolites including short-chain fatty acids, which confer a number of health-promoting benefits^22^. The role of specific amino acids on intestinal microbiota in CKD have been highlighted in a recent publication by Lobel et al.^23^ They showed that dietary sulfur-containing amino acids (i.e. methionine and cysteine) can reduce uremic toxins production. They observed that sulfide from bacterial metabolism can regulate *E. coli* indole and urea production. This occurs through an inhibition of tryptophanase by S-sulfhydration, a post translational protein modification of the cysteine residues presents in this enzyme. This dataset reinforces the role of a dietary approach to better control uremia and the need to better understand the interaction with the intestinal microbiota and the host during CKD.

In the present study, CKD mice exhibited a higher concentration of albuminemia without straightforward explanation. Only CKD mice fed with LA-AAD had a higher percentage of protein binding (%PB) of indoxyl sulfate (Supplementary Fig. 3). In the literature, albumin concentration (when in the normal range) did not influence the level of protein binding of uremic toxins, suggesting that other mechanism are involved such as post-translational modifications of plasma proteins (oxidation, carbamylation and glycosylation are for instance the most relevant processes)^24^. Also, we cannot eliminate that an increase of de novo albumin production which is probably less modified and binds better uremic toxins, could partially participate of the reduction of free fraction in CKD mice treated with LA-AAD. We can however not exclude that the reduction of free uremic toxins by LA-AAD could be, at least partially, a consequence of a structural changes in the proteins. Further studies are needed to explore this point.

Previous results, along with the present study, raise the issue that mechanisms, other than reduction in uremic toxin levels, could account for the positive effect of LPD. In an elegant experiment^25^, Vaziri & al explored the impact of urea concentration on intestinal permeability in cultured CACO-2 cells. These results were consistent with the fact that the higher blood urea nitrogen, the higher the gut barrier was permeable, leading to endotoxemia, systemic inflammation and supposedly organ fibrosis. It is widely accepted that blood urea nitrogen is directly related to protein intake. Based on this assumption, we would have expected that the LPD but not LA-AAD group could improve renal function though reduction of urea production. However, with normoproteic intakes and blood urea nitrogen levels similar to the standard diet, the LA-AAD had comparable reno-protective effects as the LPD group, challenging this view.

Renal hemodynamics variation in response to protein feeding is a well-established process, and is nowadays being explored through the renal functional reserve concept^26^. Several studies have shown that a high protein intake was associated with a higher intra-glomerular pressure, glomerular hyper-filtration and damage to glomerular structure^2,3^. Hence, the benefit of a protein-restricted diet in patients with CKD, through this hemodynamic effect, is thought to promote preservation of kidney function. Once more, our results challenge this hypothesis, given that the LA-AAD group had a comparable protein intake to the standard diet group. Amino acids, either given through stomach tube or as an intravenous perfusion, have the same renal hemodynamic effects as an acute protein ingestion, leading to renal vasodilation and GFR rise^27–29^. Different hypotheses have been discussed regarding the physiological mechanisms that may play a role in hemodynamic variations. First, amino acids could influence renal vasodilation through metabolic substrates that influence tubular sodium reabsorption or renal oxygen consumption. Secondly, humoral mechanisms have also been discussed, based on the notion that renal hemodynamic variations occurring during post-prandial state could be directly related to the release of a humoral mediator into the systemic circulation, acting on the kidney vasodilation and glomerular filtration rate. The third hypothesis regarding amino acid impact on renal hemodynamic variations focuses on intrinsic renal mechanisms, such as tubule-glomerular feedback and tubular transport^30^. It may be possible that some specific amino acids, but not all, have significant effects on renal hemodynamics^27^. In the renal proximal tubule, amino acids are co-transported with sodium, resulting in reabsorption of both through epithelial transporters. Basolateral transporters also play an important role to regulate intracellular concentration of different amino acids. Among others, the uniporter TAT1 (T-Type Amino Acid Transporter 1) is present in the small intestine and in renal proximal tubule epithelial cells. TAT1 acts as an aromatic amino acid efflux transporter and is important for the absorption of aromatic amino acids in the kidney and intestine^31^. It could therefore be interesting to measure TAT1 expression in a future study, to assess whether or not these three specific diets could have modified TAT1 expression or function.

The process by which a LA-AAD may delay renal function decline remains unclear. Tryptophan is the precursor of indoxyl sulfate, but also of serotonin and kynurenin. It has been shown that plasma tryptophan levels are decreased during CKD, proportionately to the stage of disease^32^. Accordingly, several tryptophan metabolites are also decreased in CKD patients, such as melatonin and 5-methoxytryptophan, related to a lower tryptophan hydroxylase-1 (TRP-1) expression, its main regulatory enzyme^33^. Conversely, some tryptophan metabolites are increased in CKD patients, among others IS, kynurenines, kynurenic acid and quinolonic acid^32^. Recent studies have involved tryptophan metabolites in the modulation of inflammation and fibrosis^34,35^, and anti-fibrotic therapy targeting these tryptophan metabolic by-products are currently under investigation^36^. Further experimental studies are however needed to explore whether tryptophan metabolism pathways are involved in the development of kidney fibrosis.

The mammalian target of rapamycin (mTOR) is a serine/threonine kinase which participates in several key cell functions such as cellular protein synthesis. In a 5/6 nephrectomized rat model, Ohkawa & al demonstrated that LPD and the use of mTOR inhibitor (rapamycin) had similar reno-protective effects, decreasing proteinuria and improving kidney histological damage^37,38^. Chang & al showed that 5-methoxytryptophan have beneficial effects on inflammatory mediators mediated through FoxO3a and mTOR pathway^39^. Because mTOR activity is regulated by aminoacid intake, it would be interesting to explore the hypothesis that qualitative modulation of aminoacid intake could influence mTOR pathway. Likewise, we did not analyze intestinal microbiota composition of our different groups. It might be interesting to explore the abundance and diversity of the gut microbiome, according to each specific diet.

An additional issue to consider is whether such a LA-AAD could be deleterious to the body. Indeed, tryptophan metabolism has been studied for decades by psychiatrists, as it is the precursor of serotonin, the so-called “happiness neurotransmitter”. Several animal and human experiments have been carried out to test the hypothesis of tryptophan supplementation beneficial effect on depressive symptoms, with thus far ambivalent results^40^. However, the present LA-AAD was not completely deprived in tyrosine, tryptophan and phenylalanine, and intake levels were similar to the LPD. Tryptophan and phenylalanine are essential amino acids, meaning they cannot be synthesized de novo by the human organism, and thus they must be supplied in diet. Conversely, tyrosine is considered as a conditionally essential amino acid, which means its synthesis can be limited under certain pathological conditions. Minimal dietary amounts of each aminoacid have been well established and any attempt to define a LA-AAD in human should comply with these requirements. However, current protein intakes in Western countries do bring essential aminoacids far above the minimal requirements.

We only measured the uremic toxins derivated from AAA, and therefore cannot exclude that the positive impact of LPD and LA-AAD on renal function may be related to reduction of other uremic toxins such as TMAO. LA-AAD could indeed induce a modification of the gut microbiota and reduction of TMAO production^41^. Preliminary report in CKD patients suggest that a LPD is associated with significant reduction in TMAO plasma levels after 6 months^42^. The impact of LA-AAD and LPD on TMAO production should be explored in further studies.

In conclusion the present study in CKD mice suggests that an experimental LA-AAD (i.e. low tyrosine, tryptophan and phenylalanine intake) has beneficial systemic effects, mimicking those of a LPD. Indeed, a LA-AAD appears efficient to delay renal function decline and prevent kidney fibrosis, without worsening the nutritional parameters. Significant differences between the two groups (LPD and LA-AAD) were noted such as energy intake, urea level or even inflammatory biomarkers (such as MCP-1). Future studies will be needed to determine if the specific restriction of foodstuffs containing large amounts of AAAs ( e.g.: animal meats (beef, fish), eggs, lentils,…) could improve kidney function in humans.

## Methods

### Animals

**Figure 6:**
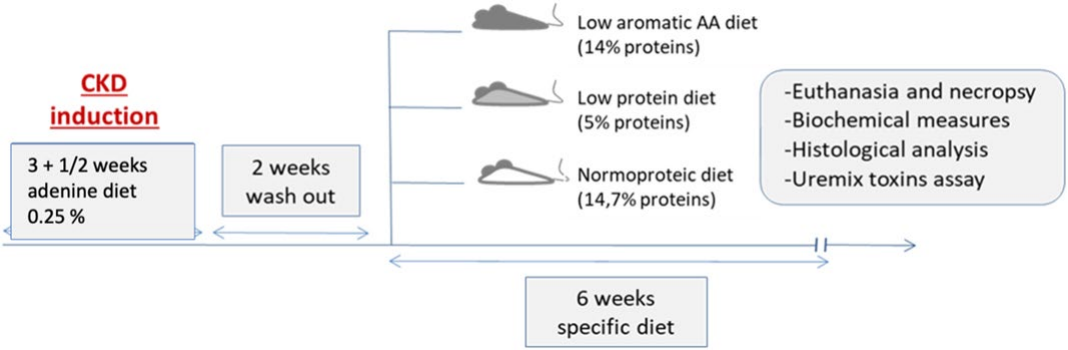
Experimental design of the study. Schematic representation of the experimental study. Half of the C57BL/6J mice were fed with an adenine diet 0.25% for 4 weeks to induce chronic kidney disease (CKD). After 2 weeks of washout, mice were divided into 6 subgroups to receive either a low protein diet, a low aromatic amino-acid diet or a normoproteic diet until terminal sacrifice after 6 weeks of specific diet.

Seventy four weeks old male C57BL/6JRj mice, were purchased from Janvier SA (Le Genest-Saint-Isle, France) and were group housed in an air-conditioned room with a controlled environment of 21 ± 0.5 °C and 60–70% humidity with a 12 h light/dark cycle. Experimental design of the study is illustrated in Fig. 6. Mice were randomly assigned in 6 groups (4–5 mice per cage). Mice were allowed a one-week period of acclimatization with free access to food and water. They were then assigned into two groups : CKD mice receiving a three weeks and a half adenine diet (0.25% w/w adenine on a A04 basis SAFE, Augy, France) to induce chronic kidney disease (CKD), and control group fed with standard diet (SAFE, Augy, France). After two week of washout, mice were divided into 6 subgroups to receive three different custom synthetic diets (SAFE, Augy, France) into each CKD and control group: LPD (5.0% proteins), a low aromatic amino-acid diet (LA-AAD) (14.0% proteins) and a normoproteic diet (NPD) (14.7% proteins) (see diet the exact composition in Table 1). Body weight and food intake were measured once a week, and food intake was calculated for each cage as the difference between the amount given and that removed from the cage. The food spillage, evaluated in preliminary experiments, was lower than 5% and therefore considered as negligible. All experimental procedures were performed in accordance with the guidelines laid down by the French Ministry of Agriculture (n°2013–118) and the European Union Council Directive for the protection of animals used for scientific purposes of September 22nd, 2010 (2010/63UE). The study protocol was approved by the local ethic committee (CETIL, Comité Ethique de l’INSA-Lyon, CNREEA n°102) on April 18th, 2018 under the reference # 11678. All experiments are conformed with the ARRIVE guidelines.

### Diuresis and 24-h proteinuria

After 6 weeks of each regimen, mice were housed for 24 h into metabolic cages (Charles River laboratories), to collect 24-h diuresis. Urine volume was determined gravimetrically, and protein concentration was measured according to the method of Bradford (Bradford reagent, Sigma Aldrich, Saint-Quentin Fallavier, France) using bovine serum albumin (BSA) as standard.

### Euthanasia and necropsy

At the end of the study, mice were euthanized with sodium pentobarbital (180 mg/kg intraperitoneally, Doletal). Body weight and body length were measured. Blood was drawn from cardiac puncture on heparinized syringe and centrifuged 4 min at 3600 × g to separate plasma. Plasma was collected, snap-frozen in liquid nitrogen and then stored at − 80 °C until analysis. Kidneys, liver, heart, gastrocnemius muscle and two different pads of white adipose tissue (epididymal and retroperitoneal) were dissected out. One kidney was stored for 48 h in a paraformaldehyde (PFA) 4% (w/v) solution for histology, and the second one was frozen in liquid nitrogen and kept at − 80 °C. All other tissues were weighted, snap-frozen in liquid nitrogen and stored at − 80 °C.

### Biochemical analysis

Plasma creatinine and urea concentrations were measured with colorimetric assays from Cayman (Ann Arbor, USA) and Sobioda (Montbonnot, France), respectively. Plasma total cholesterol and triacylglycerol concentrations were measured with colorimetric kits Cholesterol RTU and Triglycerides PAP, respectively (Biomérieux, Marcy l’Etoile, France). Plasma albumin was assessed using BCG Albumin Assay Kit (Sigma-Aldrich, St Louis, USA). To test glucose tolerance, after a 5-h fast, animals were injected i.p. with 1 g/kg D-glucose in sterile water. Blood glucose was measured prior to and 5, 10, 15, 30, 45, 60, 90, and 120 min after injection. Blood glucose values were determined from a drop of blood sampled from the tail using an automatic glucose monitor.

### Renal histology

Kidneys were paraffin-embedded, cut and stained using hematoxylin and eosin, and Sirius red staining (Cellimaps, Dijon, France). To detect Type 1 Collagen (Col-1) and CD68, formalin-fixed sections (5 μm) were deparaffinised and antigen retrieval performed by boiling sections for 20 min in 10 mM sodium citrate buffer (pH 6.0). Sections were then incubated with 10% normal horse serum followed by overnight incubation with primary antibodies rabbit anti-Col-1 (Abcam, Cambridge, UK: Anti-Collagen I antibody (ab254113)) and 1 h with rat anti-mouse CD68 (Anti-CD68 antibody, ref ab125212).

Pictures of 10X of non-overlapping fields were taken with an Olympus microscope. We used Sirius red and COL-1 staining and FIJI software to quantify the area corresponding to collagen fibrils (i.e. interstitial fibrosis) for each image. We imaged the whole kidney over three entire kidney section for each mouse, with 10 to 20 non-overlapping images with a magnification of 10 × were used. Control mice had mostly 18 images (therefore 6 by kidney section) while CKD mice rather had 10 images (3–4 by kidney section) because of the kidney size. Perivascular images were systematically excluded from the analysis. CD68 + cells number of each field were calculated and analyzed with ImageJ software (version 1.50, NIH).

To evaluate glomerular injury, images with a magnification of 400 × were used. At least 30 PAS-stained glomerular hilar cross-sections were analyzed from each kidney sample to determine glomerular area and volume using the ImageJ software. Briefly, after identifying glomeruli with both arterioles and proximal tubule in the same cross section, the outline of the glomerular area was manually outlined for the measurement. The glomerular volume VG was calculated as VG = (β/k)(A_m_)^3/2^, where β = 1.38 (shape coefficient for spheres), k = 1.1 (size distribution coefficient), and A_m_ is the surface area of the glomerulus.

### Measurement of uremic toxins

Both free and total concentrations of uremic toxins were quantified by ultra-high performance liquid chromatography with ultraviolet and fluorescence detection (UPLC-UV/FLD) as previously described^43^. Free uremic toxin concentration was measured after ultrafiltration of plasma (Molecular weight cut-off: 5 kDa). The percentage of protein binding was calculated for each toxin as.

$$ Binding \left( \% \right) = \frac{{Total\;concentration {-} Free\;concentration}}{Total\;concentration} \times 100 $$

### Analysis of gene expression

Kidney tissue was crushed into liquid nitrogen, and total RNA was extracted using TRIzol Reagent, according to the manufacturer’s instructions (Life Technologies, Carlsbad, CA, USA). Purity and concentration of RNA were determined using NanodropOne (Ozyme) and quality checked using Bioanalyser (Agilent). First-strand cDNAs were synthesized from 1 µg of total RNAs using PrimeScript RT kit (Ozyme). Real-time PCR assays were performed with Rotor-Gene 6000 (Qiagen) using SYBR qPCR Premix Ex Taq (Ozyme). TATA-box binding protein (TBP) was used as reference gene to normalize the results. For gene expression, the relative fold gene expression level for each sample was calculated using the 2^−∆∆Ct^ method. Results are expressed as the ratio of target mRNA to TBP mRNA. Primers sequences are listed in Supplementary Table 1.

### Statistical analysis

Data are expressed as mean ± SEM. All statistical analyses were performed using R version 3.5.1 and GraphPad Prism version 7.0 (GraphPad Software, La Jolla, CA, USA). QQplots and D’Agostino & Pearson test was used to assess normality. All our biological values had a normal distribution. ANOVA analyses were performed for multiple comparisons. Welch’s correction was applied in case of variance inhomogeneity. Multiple post-hoc comparisons using Dunnett test were performed to identify significant differences between groups. The different groups were compared to the CKD mice with NPD. A *p* value < 0.05 was considered as statistically significant.

## Supplementary Information

Supplementary Table 1.

Supplementary Legends.

Supplementary Figure 1.

Supplementary Figure 2.

Supplementary Figure 3.
