# Supplementary material for: A low aromatic amino-acid diet improves renal function and prevent kidney fibrosis in mice with chronic kidney disease
Source: Sci Rep. 2021 Sep 28;11:19184. doi: 10.1038/s41598-021-98718-x (PMC8479128; doi:10.1038/s41598-021-98718-x)
Supplement: Supplementary file 1 — Supplementary Table 1. [file 41598_2021_98718_MOESM1_ESM.docx]

**A low aromatic amino-acid diet improves renal function and prevent kidney fibrosis in mice with chronic kidney disease**

### Christophe Barba ^1,2^, Bérengère Benoit ^1^, Bres Emilie ^1,2^, Stéphanie Chanon^1^, Aurélie Vieille-Marchiset^1^, Claudie Pinteur^1^, Sandra Pesenti^1^, Griet Glorieux ^3^, Cécile Picard^4^, Denis Fouque ^1,2^, Christophe O. Soulage ^1$^, Laetitia Koppe ^1,2*$^

^1^ Univ. Lyon, CarMeN lab, INSA-Lyon, INSERM U1060, INRA, Université Claude Bernard Lyon 1, Rhône, Villeurbanne, FRANCE.

#### ^2^ Departement of nephrology, Hospices Civils de Lyon, Lyon Sud Hospital, Pierre Bénite, FRANCE

^3^ Nephrology Section, Department of Internal Medicine and Pediatircs, Ghent University Hospital – Ghent, BELGIUM

#### ^4^ Hospices Civils de Lyon - Centre de biologie et pathologie Est – Bron, FRANCE

|  |  |  | |  | |
| --- | --- | --- | --- | --- | --- |
| **Supplementary Table 1.**  **Sequence of primers used for qPCR analysis** | | | | | |
| **Gene name** | **Forward Primer** | | **Reverse Primer** | |  |
| *Smad3* | GGACGCAGGTTCTCCAAACC | | AAGTTCCACGGCTGCATTCC | |  |
| *Tgfb1* | AGGGCTACCATGCCAACTTC | | GTAACTGAGTTCTGACAGTG | |  |
| *Timp1* | TGGCATCTGGCATCCTCTTGT | | CATAACGCTGGTATAAGGTGG | |  |
| *Col1a1* | CAACATGGAGACAGGTCAGA | | CTTGCAGTGATAGGTGATGT | |  |
| *MCP1 (Ccl2)* | TGGAGCATCCACGTGTTGGC | | ACTACAGCTTCTTTGGGACA | |  |
| *TNF alpha* | CCAGACCCTCACACTCAGATC | | CACTTGGTGGTTTGCTACGAC | |  |
| *Interleukin 6 – IL6* | AGTTGCCTTCTTGGGACTGAT | | TCCACGATTTCCCAGAGAAC | |  |
| *Kim-1* | ACATATCGTGGAATCACAACGAC | | ACTGCTCTTCTGATAGGTGACA | |  |
| *TBP* | TGGTGTGCACAGGAGCCAAG | | TTCACATCACAGCTCCCCAC | |  |
| DNA sequences of primers used for qPCR analysis. Abbreviations: qPCR: Quantitative Polymerase Chain Reaction, Smad3: Small Mothers Against Decapentaplegic 3, Tgfb1: Transforming Growth Factor beta 1, Timp1: Tissue Inhibitor of Metalloproteinase 1, Col1a1: alpha-1 type 1 collagen, MCP1: Monocyte Chemoattractant protein 1, TNF alpha: Tumor Necrosis Factor alpha, IL-6: Interleukin 6, TBP: TATA-box binding protein, Kim-1: kidney injury molecule-1 | | | | | |
