# Supplementary material for: A low aromatic amino-acid diet improves renal function and prevent kidney fibrosis in mice with chronic kidney disease
Source: Sci Rep. 2021 Sep 28;11:19184. doi: 10.1038/s41598-021-98718-x (PMC8479128; doi:10.1038/s41598-021-98718-x)
Supplement: Supplementary file 2 — Supplementary Legends. [file 41598_2021_98718_MOESM2_ESM.docx]

**Supplementary Figure 1: Gene expression of tubular cell damage markers in kidney**

Effects of normoproteic diet (NPD), low protein diet (LPD) and low aromatic amino-acid diet (LA-AAD) on relative mRNA expression of Kim-1 (kidney injury molecule-1). TBP (TATA-Box Binding Protein) was used as reference gene to normalize the results. Data are expressed as mean ± SEM for n = 6-8 animals in each group. *p < 0.05, ***p < 0.001 vs CKD-NPD; (ANOVA and Dunnett post hoc test).

**Supplementary Figure 2: Gene expression of tubular cell damage markers in kidney**

(A) Blood glucose measured and (B) AUC during an i.p. glucose tolerance test (i.p.GTT, 1 g/kg D-glucose) in control and CKD mice fed with normoproteic diet (NPD), low protein diet (LPD) or low aromatic amino-acid diet (LA-AAD). Data are expressed as mean ± SEM for n = 7 animals in each group . **P < 0.01 (ANOVA and Dunett post hoc test).

**Supplementary Figure 3: Percentage of protein binding (%PB) of (A) Indoxyl Sulfate (IS) and (B) p-cresyl sulfate (PCS) in control and CKD mice**

Data are expressed as mean ± SEM for N= 6-11 animals in each group. *p < 0.05, vs CKD-NPD; (ANOVA and Dunnett post hoc test). CKD: chronic kidney disease; NPD: normoproteic diet, LPD: low protein diet; LA-AAD: low aromatic amino-acid diet (LA-AAD). Data are expressed as mean ± SEM for n = 6-11 animals in each group. *p < 0.05, ***p < 0.001 vs CKD-NPD; (ANOVA and Dunnett post hoc test).
