# Supplementary figures and images for: A low aromatic amino-acid diet improves renal function and prevent kidney fibrosis in mice with chronic kidney disease
Source: Sci Rep. 2021 Sep 28;11:19184. doi: 10.1038/s41598-021-98718-x (PMC8479128; doi:10.1038/s41598-021-98718-x)

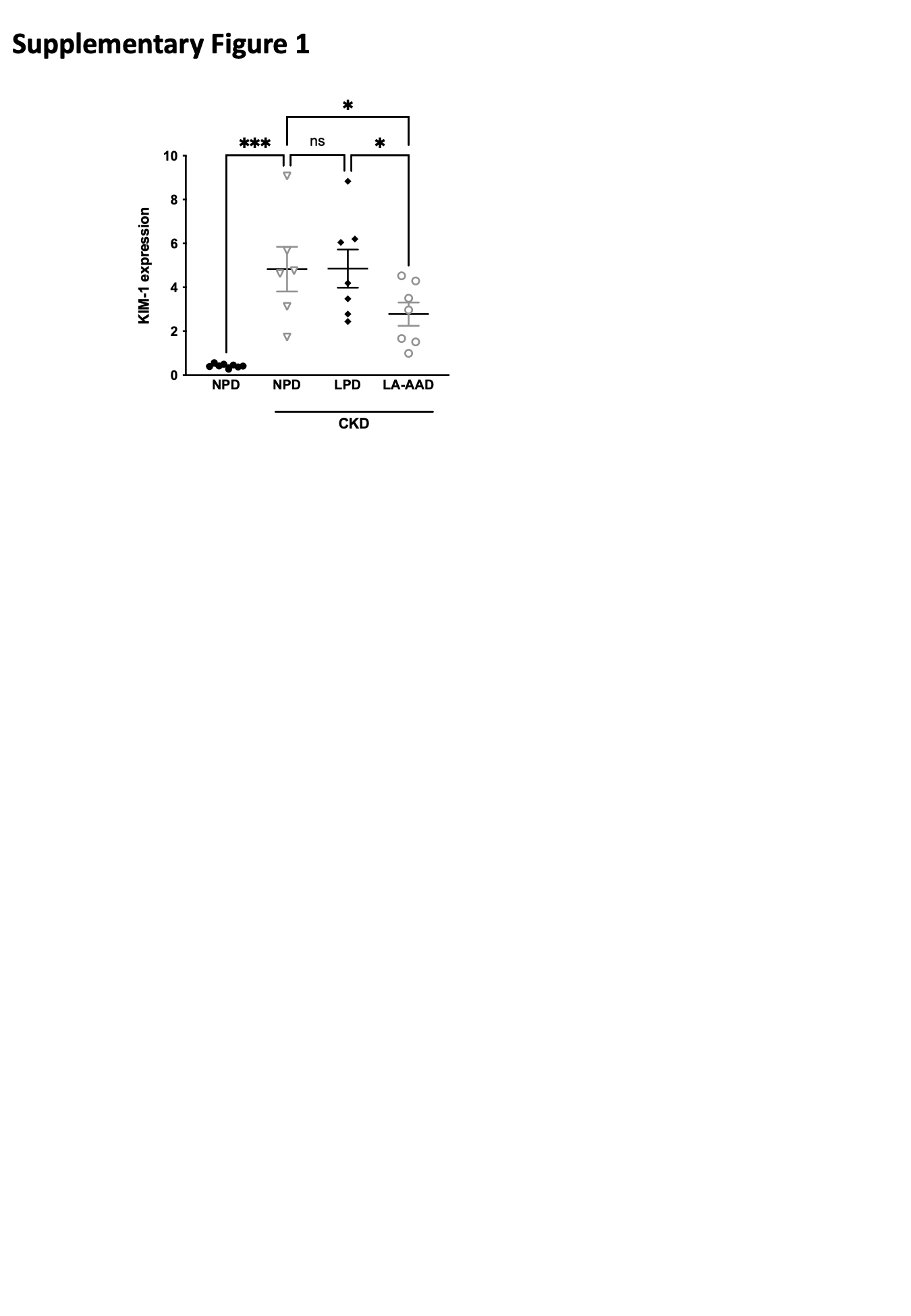

Supplement: Supplementary file 3 — Supplementary Figure 1. [file 41598_2021_98718_MOESM3_ESM.tiff]

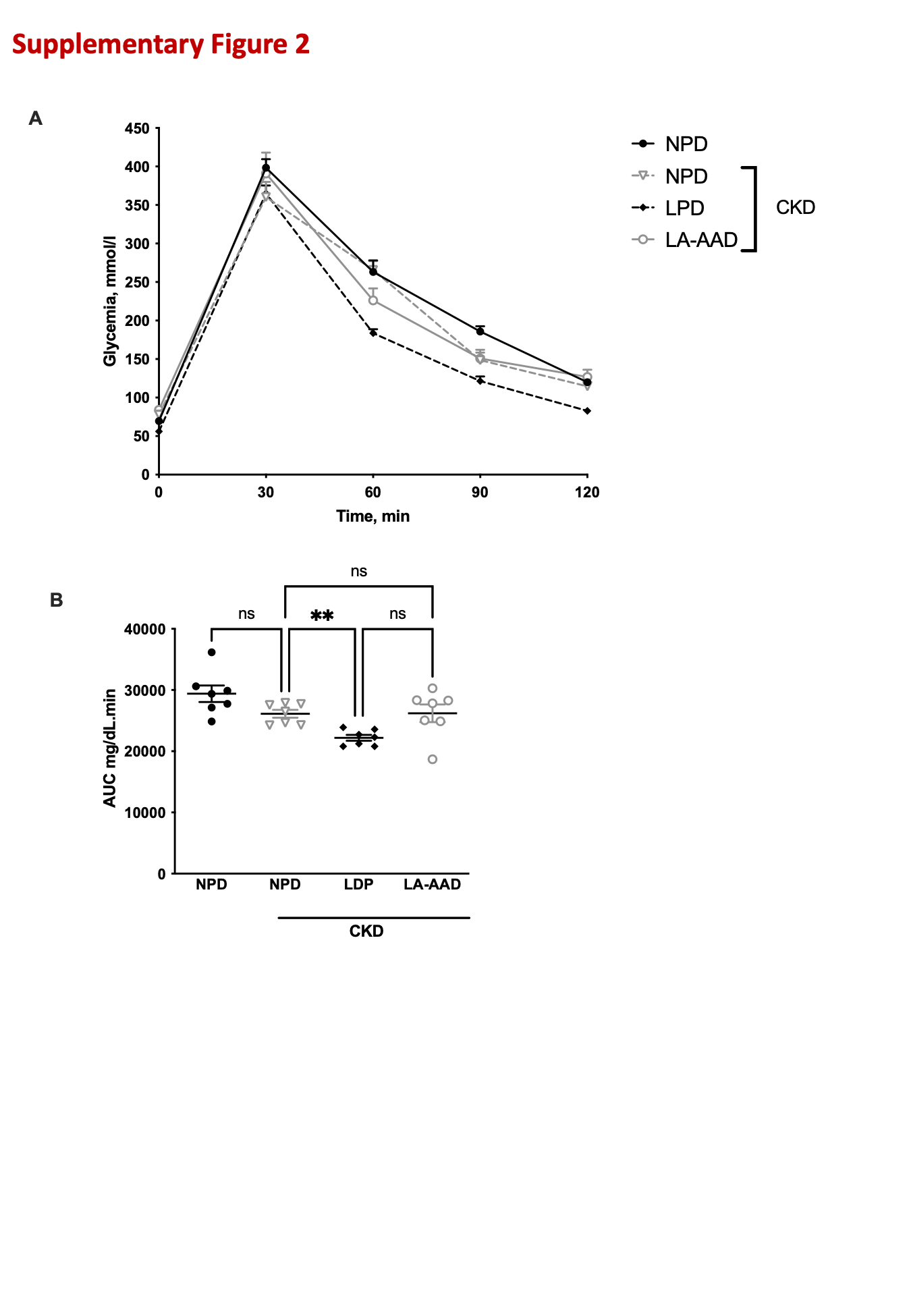

Supplement: Supplementary file 4 — Supplementary Figure 2. [file 41598_2021_98718_MOESM4_ESM.tiff]

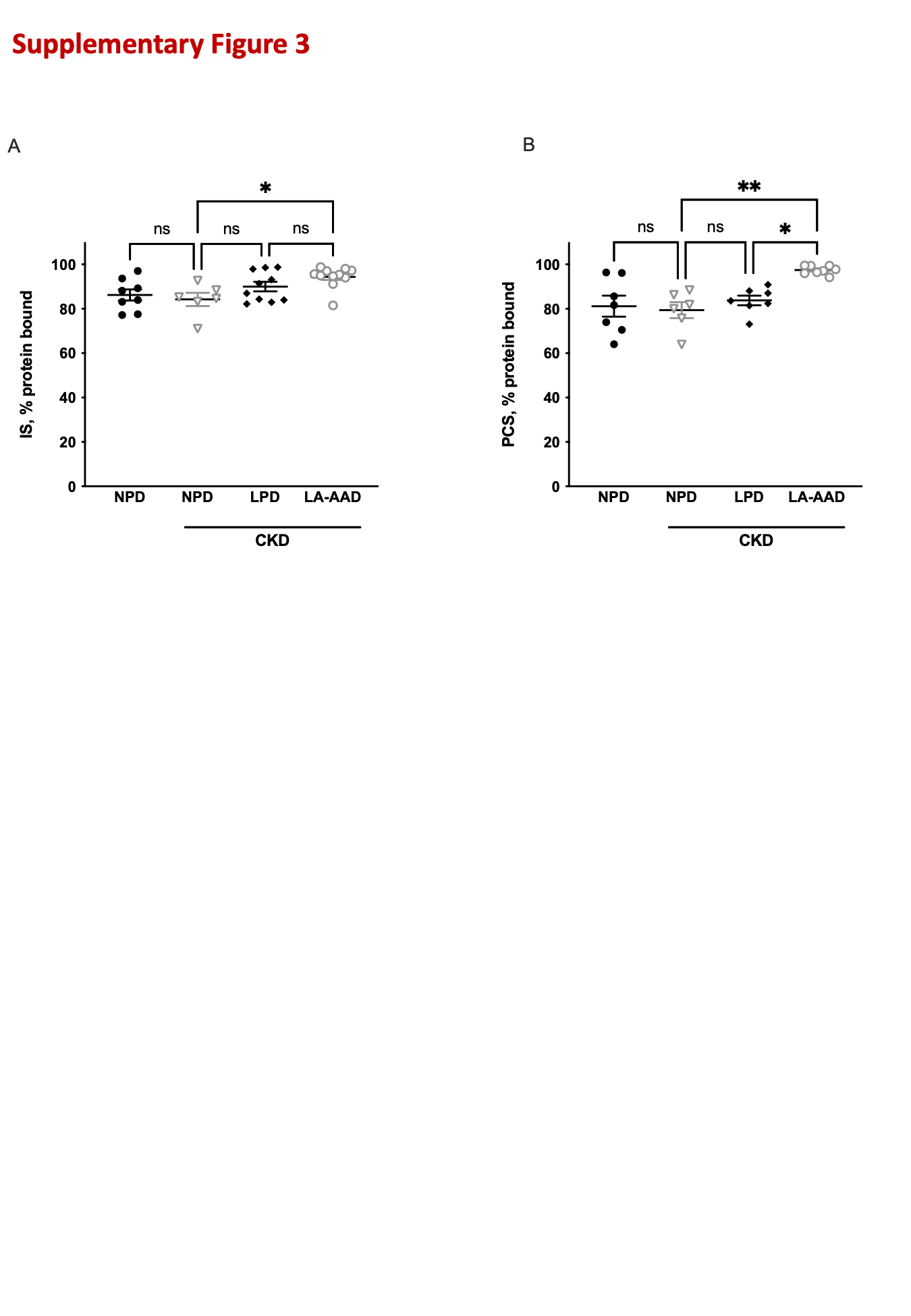

Supplement: Supplementary file 5 — Supplementary Figure 3. [file 41598_2021_98718_MOESM5_ESM.tiff]
